# Association between maxillary sinus pathologies and healthy teeth^☆^^☆☆^

**DOI:** 10.1016/j.bjorl.2015.11.004

**Published:** 2015-12-10

**Authors:** Gina D. Roque-Torres, Laura Ricardina Ramirez-Sotelo, Sergio Lins de Azevedo Vaz, Solange Maria de Almeida de Bóscolo, Frab Norberto Bóscolo

**Affiliations:** a Department of Oral Diagnosis, Faculdade de Odontologia de Piracicaba, Universidade Estadual de Campinas (UNICAMP), Piracicaba, SP, Brazil; b Dental Radiology, Universidade Estadual de Campinas (UNICAMP), Piracicaba, SP, Brazil; c Department of Dental Clinic, School of Dentistry, Universidade Federal do Espírito Santo (UFES), Vitória, ES, Brazil

**Keywords:** Molar tooth, Premolar tooth, Maxillary sinus, Dental root, Cone beam computed tomography, Dente molar, Dente pré-molar, Seio maxilar, Raiz dentária, Tomografia computadorizada de feixe cônico

## Abstract

**Introduction:**

The proximity of the roots to the maxillary sinus can create a variety of risks.

**Objective:**

To evaluate the relationship between the roots of healthy teeth and the maxillary sinus, as well as the occurrence of sinus pathologies.

**Methods:**

Three radiologists analyzed 109 cone beam computed tomography (CBCT) images. The Kappa test was used to assess the intra- and inter-rater agreement. The chi-squared test and prevalence ratio were used to test the hypothesis that roots of healthy teeth in the maxillary sinus favored the occurrence of sinus pathologies (*p* = 0.01).

**Results:**

Intra- and inter-rater agreement ranged from good to excellent. The chi-squared test demonstrated a statistically significant difference (*p* = 0.006) between the tooth roots in diseased maxillary sinuses (6.09%) and those in normal sinuses (3.43%). The prevalence ratio test showed a statistically significant higher prevalence of tooth roots in diseased sinuses than in normal sinuses (*p* < 0.0001). Roots in the maxillary sinus were 1.82 times more associated with diseased sinuses.

**Conclusion:**

Dental roots in the maxillary sinus are almost twice as likely to be associated with diseased sinuses than normal sinuses. Healthy teeth whose roots are inside the maxillary sinus may induce an inflammatory response in the sinus membrane. It is suspected that dental procedures may exacerbate the condition.

## Resumo

**Introdução:**

A proximidade das raízes com o seio maxilar pode criar uma variedade de riscos.

**Objetivo:**

Avaliar a relação entre as raízes dos dentes hígidos com o seio maxilar, assim como com a ocorrência de doenças sinusais.

**Método:**

Três radiologistas analisaram 109 imagens de tomografia computadorizada de feixe cônico (TCFC). O teste Kappa avaliou a concordância intra e interexaminadores. Os testes de Qui-quadrado e Razão de Prevalência foram utilizados para testar a hipótese de que raízes de dentes hígidos dentro do seio maxilar favorecem a ocorrência doenças sinusais (nível de significância = 0,01).

**Resultados:**

A concordância intra e interexaminadores variou de boa a excelente. O teste Qui-quadrado mostrou uma diferença estatisticamente significante (p  =  0,006) entre as raízes dentárias dentro do seio maxilar patológico (6,09%) e aquelas dentro do seio normal (3,43%). O teste de Razão de Prevalência mostrou uma ocorrência de raízes dentárias dentro de seios patológicos estatisticamente maior do que dentro de seios normais (p  <  0,0001). As raízes dentro do seio maxilar foram encontradas 1,82 vezes mais associadas a seios patológicos.

**Conclusão:**

Raízes dentárias dentro do seio maxilar são quase duas vezes mais associadas a seios patológicos do que a seios normais. Dentes hígidos que têm raízes dentro do seio maxilar podem induzir uma resposta inflamatória da mucosa sinusal. Suspeita-se que procedimentos odontológicos possam agravar tal situação.

## Introduction

Maxillary sinuses can vary in size and shape from one individual to another, or even between the right and left sides in the same individual. In approximately half of the population, their length also varies. The floor of the maxillary sinus extends into the alveolar process between the roots of adjacent teeth, creating elevations and depressions called “extensions,” with narrow cortical areas.^1^, ^2^ Through histological sections, it has been radiographically demonstrated that most of the roots projecting into the maxillary sinus were in fact surrounded by a thin layer of cortical bone, with perforations present in 14–28% of cases.^3^ Under normal conditions, the relationship between the tooth and the floor of the maxillary sinus consists of either a thin layer of compact bone that provides support to the apical periodontal ligament fibers, to which it firmly adheres, or there is a direct relationship with the maxillary sinus mucosa. The inner lining of the maxillary sinus cavity is devoid of periosteum; therefore, in the absence of a thin layer of intervening bone, the periodontal tissues are in direct contact with the basal surface of the sinus mucosa.^2^

The roots of the upper premolars, molars, and occasionally the canine teeth have a close relationship with the maxillary sinus; in some cases, they may even protrude into it.^4^, ^5^, ^6^ It has been demonstrated that the closer the tooth apex is to the floor of the maxillary sinus, the greater the impact on antral tissue.^7^ This relationship can result in a variety of risks, especially for certain surgical procedures, such as tooth extraction and implant placement, or during endodontic or orthodontic treatments.^4^, ^5^, ^8^ An accurate description of the relationship between the apices of the upper teeth and the lower wall of the maxillary sinus, as well as the thickness of the cortical bone between these structures, is essential for planning dental procedures.

Dental radiographs, such as panoramic radiography, consist of two-dimensional images and, as such, are inappropriate and/or of little use for accurate morphometric assessment of bone relationships.^4^ In cases where the panoramic radiograph reveals a possible relationship between a tooth that has undergone intervention and its contact with the adjacent maxillary sinus, evaluation by cone beam computed tomography (CBCT) can assist in dental treatment planning. This imaging modality allows for a thorough analysis of the anatomical relationship between the maxillary sinus and the roots of the upper teeth,^4^, ^6^, ^9^, ^10^ thus overcoming the limitations of panoramic radiography, providing multiplanar views without magnification, distortion, or superimposition.^11^ This technique is superior to multi-slice computed tomography due to its higher image resolution, reduced radiation exposure, and lower equipment cost.^12^

In apical periodontitis, a periodontal disease,^13^, ^14^ treatment with implants and extraction of upper premolars and molars^15^ may increase the risk of pathological processes in the adjacent maxillary sinus. Of the odontogenic sinus diseases, apical periodontitis and periodontal disease account for 83% of all cases having dental origin.^13^, ^16^ The most frequent maxillary sinus pathologies are sinus mucosal thickening and mucous retention cysts, with a prevalence ranging from 8% to 29% and 2% to 36%, respectively.^16^, ^17^, ^18^, ^19^ Another study has reported a prevalence rate of odontogenic maxillary sinusitis ranging from 10% to 86%.^20^

There are few studies describing the relationship between maxillary sinus diseases and healthy upper posterior teeth in contact with the maxillary sinus. Therefore, in order to answer this question, this study aimed to evaluate the relationship between the roots of healthy teeth with healthy and diseased (mucosal thickening and mucous retention cysts) maxillary sinuses through CBCT. This research can help identify correlations between teeth and sinus diseases without causal factors.

## Methods

For this study, 109 CBCT images were selected, obtained independently of this research for diagnosis and treatment planning purposes. All images belonged to the digital archive of the Radiology Clinic. After approval of the study protocol by the local Research Ethics Committee (Protocol No. 084/2012), the sample consisted of 78 women and 31 men, mean age 22 years (range: 18–30 years), without distinction by race, gender, social class, or other socioeconomic characteristics. In all images, both maxillary sinuses (*n* = 218) and the roots of all premolars and molars on both sides (*n* = 1875) were evaluated.

CT scans were obtained using the I-CAT Classic scanner (Imaging Sciences International – Pennsylvania, United States), using the following exposure parameters: 8 mA, 120 kVp, acquisition time of 40 s, 13 cm × 17 cm field of vision, and voxel size of 0.25 mm. Images with good sharpness, density, and contrast were selected. The images had to present the apices of the upper posterior teeth and the maxillary sinus along their entire length.

For inclusion in the study, the images had to present complete permanent dentition, with no evidence of any type of dental pathology, with fully formed root apexes, and without the presence of supernumerary teeth. Most reasons for CBCT imaging comprised temporomandibular joint disorder, orthodontic treatment, and evaluation of third molars. Images with evidence of carious lesions, prosthetic crowns, filled root canal, periapical lesions, bifurcation lesions, and moderate or severe bone loss were excluded.

**Figure 1:**
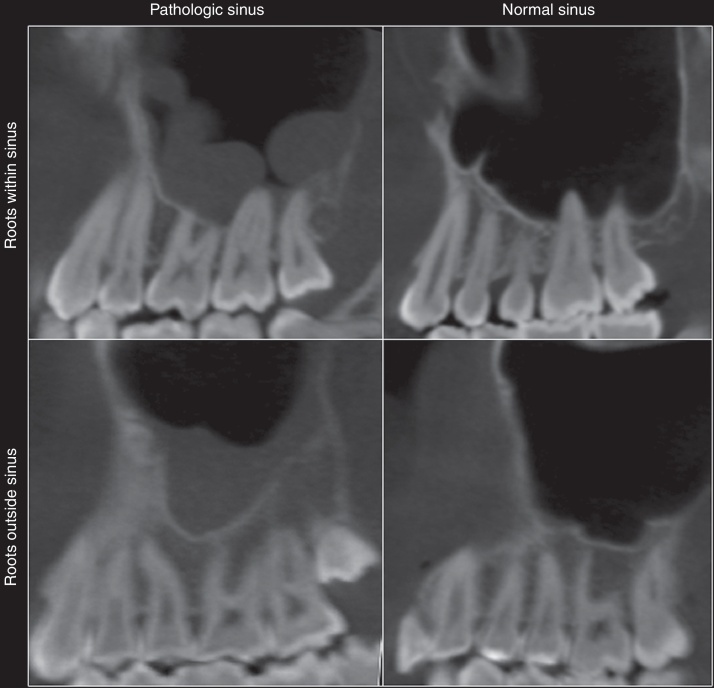
The sinus cavities were classified as diseased or normal. The tooth roots were considered to be inside or outside the sinus.

Three experts in dental radiology, who had a minimum experience of two years in CBCT, were selected for the study. In low light conditions, these experts independently assessed the presence or absence of sinus disorders (mucosal thickening or mucous retention cysts), and the topographical relationship between the maxillary sinus and each apex of the upper posterior teeth (first and second premolars, as well as first and second molars). Only these teeth were assessed, as their root apexes are the closest to the maxillary sinus floor. The relationship of the root with the maxillary sinus was determined as follows: roots in the maxillary sinus, and roots outside of the maxillary sinus (apexes in contact or not with the limits of the maxillary sinus cortical). This topographical evaluation (Fig. 1) of the teeth was performed using multiplanar reconstruction (MPR). All views attainable within the software were made available for the detection of pathological findings; thus, the evaluators were allowed to adjust the brightness, contrast, and zoom of the image. The use of filters was not authorized.

The mode was obtained from the three evaluations, both for the topographic relationship between the root and the maxillary sinuses and for the presence or absence of sinus pathology. The frequency of tooth roots inside and outside of the maxillary sinus and the presence or absence of pathology in the maxillary sinus were quantified and subsequently assessed with the chi-squared test and prevalence ratio. A *p*-value < 0.01 was considered as statistically significant.

Thirty days after the first evaluation, 27% of the sample was re-evaluated for reliability measurements. Inter- and intra-rater correlation was calculated using the Kappa test (poor agreement, 0.40; moderate agreement, 0.40–0.59; good agreement, 0.60–0.74; excellent agreement, 0.75–1.00).

## Results

The Kappa values for inter- and intra-rater agreement in the assessments of root-maxillary sinus relationship and of sinus disorders ranged from good to excellent (Table 1).

**Table 1:** Kappa values for inter- and intra-rater agreement.

	Rater 1	Rater 2	Rater 3
*Root-maxillary sinus relationship*			
Rater 1	0.65	–	–
Rater 2	0.61	0.62	0.61
Rater 3	0.62	–	0.63

*Sinus pathology*			
Rater 1	1.00	–	–
Rater 2	0.61	1.00	0.78
Rater 3	0.66	–	0.93

The frequency of diseased maxillary sinus (34.98%) was lower than that of normal maxillary sinuses (65.02%). Most roots were located outside the maxillary sinus (95.63%). This was observed in normal maxillary sinuses (96.57%), as well as in diseased sinuses (93.91%; Table 2).

**Table 2:** Number of roots inside and outside diseased and normal maxillary sinuses.

	Diseased sinus, *n* (%)	Normal sinus, *n* (%)	Total, *n* (%)
Roots outside the maxillary sinuses	632 (93.91)	1208 (96.57)	1840 (95.63)
Roots inside the maxillary sinuses	41 (6.09)	43 (3.43)	84 (4.37)
Total	673	1251	1924

*χ*^2^ = 7387; *p* = 0.006.

The chi-squared test demonstrated that there was a statistically significant difference (*p* = 0.006) between the occurrence of tooth roots within diseased maxillary sinuses (6.09%) and within normal sinuses (3.43%). A statistically greater difference was observed in the prevalence of tooth roots within diseased sinuses than in normal sinuses (*p* < 0.0001). Roots located within the maxillary sinuses were found to be present 1.82 times more frequently in diseased sinuses (95% confidence interval: 1.67–1.98).

The stratification of teeth group with the diseased sinuses, in increasing order, was as follows: first premolar (0%), lower second premolar (7.31%), first molar (41.46%), and second molar (51.21%). The order for normal sinus was as follows: first premolar (4.65%), second premolar (6.97%), second molar (37.2%), and first molar (51.16%; Table 3).

**Table 3:** Number of roots inside diseased and normal maxillary sinuses, by tooth group.

	Diseased sinus, *n* (%)	Normal sinus, *n* (%)	Total, *n* (%)
Second molar	21 (51.21)	16 (37.2)	37 (44.04)
First molar	17 (41.46)	22 (51.16)	39 (46.42)
Second premolar	3 (7.31)	3 (6.97)	6 (7.14)
First premolar	0 (0)	2 (4.65)	2 (2.38)
Total	41 (100)	43 (100)	84 (100)

## Discussion

This study aimed to investigate the relationship between tooth roots in the maxillary sinuses and the presence of sinus diseases. The patients in this sample did not present dental diseases such as caries, periapical lesions, filled root canals, or significant alveolar bone loss.

The topographic root-maxillary sinus relationship was assessed using CBCT images obtained from an archive. Studies have shown that when panoramic radiograph was used as a method of assessment, the root-maxillary sinus relationship was improperly determined in 39–57% of cases.^5^, ^21^ The literature has also shown that the reliability in detecting sinus diseases, such as mucosal thickening, is higher using CBCT than 2-D X-rays.^22^ Consequently, CBCT was considered to be a reliable method for the purposes of the present study.

Three experts in dental radiology, who had at least two years experience with CBCT images, evaluated the images of this study independently; the mode of the three evaluations was then obtained. The inter-rater agreement was measured by the Kappa test. This methodology is rarely applied in similar studies, which often use only one evaluator,^12^, ^20^, ^23^ or two evaluators who then reach a consensus,^22^, ^24^, ^25^ and do not present statistical data regarding the agreement between evaluators. In this study, intra- and inter-rater agreements regarding the tooth-maxillary sinus relationship were good. For sinus conditions, the inter-rater agreement was also good, but for raters 2 and 3, it was excellent. The differences found in this aspect of the study may be related to the fact that the experts were free to evaluate the MPR images as preferred. This may also reflect the difficulty in visualizing thin alveolar cortical plates in the region where the roots were in contact with the maxillary sinus. For future studies, it is recommended that the evaluation of MPR images should be standardized; however, still images should not be used, as CBCT requires a dynamic assessment, comprising all cuts.

Mucosal thickening and mucous retention cysts were grouped as diseased sinuses. The sinus mucosa is considered as thickened when the membrane is 2–6 mm thick. The etiological factors are related to some type of irritation, such as odontogenic cysts or allergy.^19^, ^26^, ^27^, ^28^, ^29^, ^30^, ^31^ Light mucosal thickening of the maxillary sinus is a normal finding in asymptomatic patients,^32^ but a thickening greater than 2 mm can be associated with maxillary sinusitis.^33^ Using previous studies as reference, mucosae at least 3 mm thick were considered as thickened in the present study.^26^, ^34^ Mucous retention cysts are bodies that develop as a result of a blockage of the sinus ostium and usually resolve spontaneously. They are common findings in CBCT images, but cannot be detected without proper training in dental radiology.^35^ Although they are usually present in asymptomatic patients, it is important to disclose them in CBCT image reports.

The topographic relationship between tooth roots and the maxillary sinus has been studied in the literature. In one study,^4^ the authors reported that two of the 38 study subjects (5%) had roots that protruded into the sinus cavity. A similar rate (10%) was observed in another study.^6^ Therefore, the incidence of tooth roots in the maxillary sinus in the present study is in agreement with the literature.^4^, ^6^ Oral surgeons should be aware of the amount of bone around the maxillary sinus, so that the necessary precautions can be taken to avoid perforation of the sinus membrane and introduction of foreign bodies into the maxillary sinus during dental treatments.^3^, ^36^

The literature has shown that, due to the close relationship between the teeth and the floor of the maxillary sinus, dental infections can extend to the maxillary sinus.^24^ Direct contact between periodontal tissues and sinus mucosa can occur because of the proximity of the maxillary sinus and the upper posterior teeth implanted in the alveolar process.

Maxillary sinus infections and mucosal thickening have been identified in 2% of patients with dentulous superior maxillas.^18^ However, these authors did not find mucous retention cysts in edentulous patients, which may suggest an odontogenic etiology for this condition. In another study,^37^ 75% of cases with maxillary sinusitis were associated with dental conditions. Other studies have shown that periapical lesions^19^, ^38^ and periodontal disease^12^, ^19^, ^23^, ^24^, ^39^ are associated with sinus pathologies. It has been demonstrated that, in cases of apical periodontitis, when the tip of the tooth root was in contact with the floor of the maxillary sinus, the incidence of mucosal thickening was lower than when the tip of the root exceeded the floor of the maxillary sinus.^25^

Of all the groups of teeth, the tomographic distance between the upper second molar root apex and the maxillary sinus floor is reported to be the smallest among all maxillary posterior teeth.^4^, ^40^ In line with these reports, in the present study the group of teeth with the most dental roots within diseased sinus was the second molar. Likewise, the first molar had more roots inside normal maxillary sinuses, although this number was quite close to that of the second molar.

Among the limitations of this study, the lack of clinical data on patients evaluated by CBCT is noteworthy. It is also important to highlight that we did not analyze the histological samples that more accurately determine the different pathological changes in the maxillary sinus tissue. The dental radiologists who evaluated the CBCT images were free to adjust the software, so there was little standardization in the assessment of the images.

## Conclusions

In the studied sample, tooth roots within the maxillary sinus were almost twice as likely to be associated with pathological maxillary sinuses than normal maxillary sinuses.

## Conflicts of interest

The authors declare no conflicts of interest.
